# Empowered Relief vs Cognitive Behavioral Therapy for Patients With Systemic Lupus Erythematosus and Chronic Pain: Study Protocol for a National, Randomized, Noninferiority Trial

**DOI:** 10.2196/100104

**Published:** 2026-08-25

**Authors:** Titilola Falasinnu, Arayam Y Hailu, Emma Adair Monson, Jessica Clifton, Calia Torres, Troy C Dildine, Brittany Dorsonne, Elizabeth Heggan, Gabrielle Riazi, Kristen Slater, Heather Poupore-King, Luzmercy Perez, James Hellewell, Lu Tian, Steve Denton, Andrea DeVries, Jackie Miefert, Sean C Mackey, Matthias Cheung, Beth D Darnall

**Affiliations:** 1 Division of Immunology and Rheumatology Stanford University School of Medicine Palo Alto, CA United States; 2 Department of Anesthesiology, Perioperative and Pain Medicine Division of Pain Medicine Stanford University School of Medicine Palo Alto, CA United States; 3 Parhelia Wellness Santa Rosa, CA United States; 4 Heersink School of Medicine University of Alabama Birmingham, AL United States; 5 Department of Psychiatry Lehigh Valley Health Network part of Jefferson Health Allentown, PA United States; 6 Health Care Services SCAN Health Plan Long Beach, CA United States; 7 Family Medicine Intermountain Health Layton, UT United States; 8 Department of Biomedical Data Science and (by courtesy) Statistics Stanford University School of Medicine Palo Alto, CA United States; 9 Real-World Evidence Institutional Partnerships Humana Healthcare Research, Inc Louisville, KY United States; 10 Department of Pharmacy Practice Thomas J Long School of Pharmacy University of the Pacific Stockton, CA United States; 11 Department of Anesthesiology, Perioperative and Pain Medicine Division of Pain Medicine, Stanford Pain Relief Innovations Lab Stanford University School of Medicine Palo Alto, CA United States

**Keywords:** systemic lupus erythematosus, SLE, digital health, health equity, patient centered, online, telehealth, skill based, psychology

## Abstract

**Background:**

Systemic lupus erythematosus (SLE) presents substantial life challenges, reflecting the complex and heterogeneous nature of the disease. Pain is common and often one of the earliest manifestations. Skill-based behavioral pain treatments are often inaccessible to patients with SLE, and no SLE-specific evidence exists to guide patient and clinician treatment choices. To fill a pressing evidence gap for SLE pain treatment, the Lupus PROGRESS (Pain Relief With Online Groups That Empower Skill-Based Symptom Reduction) trial will compare a lower-burden evidence-based behavioral pain treatment with online multisession cognitive behavioral therapy (CBT; the gold standard behavioral treatment for chronic pain) while also enhancing diversity and representativeness among patient advisors and study participants.

**Objective:**

Lupus PROGRESS is a national, pragmatic, randomized noninferiority trial comparing the effectiveness of 2 evidence-based behavioral pain treatments (online 8-session CBT vs 1 session the Empowered Relief program; Stanford University) in adults with SLE and chronic pain in the United States for reducing pain intensity and pain interference at 3 months after treatment (multiple primary end points).

**Methods:**

In total, 150 adults with SLE and pain (≥3 months, intensity of ≥3/10) will be enrolled from academic medicine, community health network, and insurance payer study sites. Participants are randomized 1:1 to either 1-session Empowered Relief or 8-session CBT, with both treatments delivered online to groups. Mixed-model repeated measures and intention-to-treat analytic approaches will be used to test comparative effectiveness aims from baseline to 3 months after treatment (the primary end point). Multiple primary outcomes are pain intensity and pain interference; priority secondary outcomes are sleep disturbance, pain catastrophizing, pain bothersomeness, and anxiety; and other secondary outcomes are satisfaction with roles and responsibilities and global impression of change, anger, fatigue, and depression. Durability of effects will be tested at 6 months after treatment. Empowered Relief is hypothesized to have superior treatment completion and adherence and lower treatment burden ratings than CBT. In exploratory analyses, we will compare health care use through medical record and claims data 3 months prior to enrollment to the last 3 study months, hypothesizing reduced noninferiority in reduced use. The study also incorporates a multilevel patient engagement infrastructure to improve accessibility, recruitment, and study relevance.

**Results:**

As of May 2026, we have enrolled 130 participants. Enrollment is anticipated to end in November 2026, with treatments completed by April 2027 and full analyses completed by February 2028.

**Conclusions:**

Lupus PROGRESS will provide real-world pragmatic evidence on whether a 1-session online group skill-based treatment is noninferior to 8-session CBT. Results from this patient-centered study will inform more accessible, equitable, and patient-centered chronic pain care for people living with lupus.

**Trial Registration:**

ClinicalTrials.gov NCT05612750; https://clinicaltrials.gov/study/NCT05612750

**International Registered Report Identifier (IRRID):**

DERR1-10.2196/100104

## Introduction

Systemic lupus erythematosus (SLE) is a rare chronic inflammatory rheumatic disease with multiple organ involvement. SLE disproportionately affects young women, including approximately 200,000 Americans [1], and is the seventh most common cause of death among female individuals aged 15 to 24 years in the United States [2]. Pain burden in SLE is substantial, with US data showing that approximately half of patients with SLE having overlapping chronic pain conditions, including low back pain, migraines, and fibromyalgia [3]. Patients with SLE often face significant challenges in managing their pain and distress and report little relief [4]. Psychological distress is common in SLE [5], exacerbated by untreated pain [6], and is linked to increased SLE activity [7]. Recent real-world evidence demonstrates that SLE pain treatment is characterized by high prescribing intensity and substantial polypharmacy, with approximately half of patients (and 70% of young adults) cycling through 5 or more pain medication prescriptions within a year [8]. Despite recent shifts away from high-risk treatments, such as opioids and glucocorticoids, treatment pathways vary widely by demographic factors, underscoring persistent gaps in effective and equitable pain care.

Behavioral pain treatments are low risk and help patients acquire strategies and skills to support long-term pain and symptom management. In the general non-SLE chronic pain behavioral treatment literature, 8-session cognitive behavioral therapy (CBT), comprising 16 hours of therapist-delivered treatment over 2 months, is considered the gold standard and best studied skill-based intervention for reducing pain and other symptoms [9-11]. Specific to adults with SLE, the randomized studies on behavioral pain treatments are sparse. We identified 3 SLE single-site randomized controlled trials, all with relatively small sample sizes, consistently reporting that multisession CBT improved quality of life and reduced psychological symptoms [12-14]. Evidence is lacking for multisite SLE studies with larger, diverse, and representative study populations [15].

While effective, the feasibility of receiving CBT is often limited by a variety of factors, including insurance coverage and treatment costs, therapist availability, and the treatment time burden [16]. As a 1-session pain relief skill intervention, the Empowered Relief program (Stanford University) may provide greater access and reach, particularly for underserved populations [17]. Evidence from multiple randomized trials has demonstrated the efficacy of Empowered Relief for reducing pain and multidimensional symptoms in patients with chronic back pain [9,18] and of online-delivered Empowered Relief in patients with mixed-etiology chronic pain [19,20] and in rare connective tissue disorders [21]; Empowered Relief is untested in SLE. A prior randomized trial showed that Empowered Relief was noninferior to CBT for reducing chronic low back pain and a range of symptoms at 3 months after treatment, thus providing a strong foundation for the current study [18].

The Lupus PROGRESS (Pain Relief With Online Groups That Empower Skill-Based Symptom Reduction) study extends this prior work and will test the noninferiority of 1-session Empowered Relief vs 8-session CBT specifically in patients with SLE, with online delivery of both treatments giving greater reach and home-based access to diverse patients. Lupus PROGRESS builds on a separate national parent randomized trial being conducted among 1200 people with chronic pain of all types [22]. Lupus PROGRESS will generate comparative effectiveness evidence and yield important and specific insights to guide the decision-making of patients with SLE and clinicians and enhance care delivery for this underserved patient population.

## Methods

### Study Setting and Recruitment

Lupus PROGRESS is enrolling 150 individuals with SLE and chronic pain from 3 study sites. Consistent with the pragmatic design, enrollment reflects site-specific patient populations and recruitment capacity: Humana Healthcare Research is part of the national Medicare Advantage plan (anticipated n=120), Stanford Medicine is an academic medical center (anticipated n=15), and Intermountain Health is an integrated care network (anticipated n=15). Recruitment at Humana involves mailed advertising sent to individuals identified through database diagnostic codes (*International Classification of Diseases*). Stanford recruitment involves direct-to-participant email outreach (n=645) through an opt-in institutional pain research registry where patients self-identify as having SLE. Intermountain Health recruits patients with SLE via study advertisements mailed to patients with chronic pain–related *International Classification of Diseases* codes.

### Inclusion and Exclusion Criteria

Inclusion criteria are age ≥18 years, receipt of medical care through a Lupus PROGRESS study site, experience of chronic pain for 3 or more months with past-month average severity of 3 or more on a 10-point scale [23], and diagnosis of SLE. To enhance SLE diagnostic accuracy, we are applying criteria based on the work by Falasinnu et al [23] to increase the likelihood of true idiopathic SLE rather than drug-induced lupus or other secondary lupus conditions more common in older adults and to align with typical SLE onset and disease patterns. Patients must be actively and continuously enrolled in a commercial or Medicare Advantage plan for at least 6 months to ensure that all outcome measures are available. Following the work by Falasinnu et al [3], patients are required to have 2 or more lupus diagnostic codes from Textbox 1 on different dates separated by 7 to 365 days during the data period, with at least one code from a rheumatologist, dermatologist, or nephrologist. Per Humana institutional policies, members enrolled in administrative services–only insurance, Medicaid, and other research-excluded groups will be excluded. Other exclusion criteria are inability to consent, complete study measures (online or paper based), or participate in internet-based treatment groups. Past-3-month receipt of Empowered Relief or CBT and active suicidal ideation at screening are also exclusionary. Concomitant pain care of other types is allowed, self-reported throughout the trial, and will be reported in the study.

**Lupus International Classification of Diseases codes (2 required for eligibility).**

- M32.9: systemic lupus erythematosus, unspecified
- M32.10: systemic lupus erythematosus, organ or system involvement unspecified
- M32.11-M32.15: systemic lupus erythematosus, specific to organ involvement
- M32.19: systemic lupus erythematosus, other organ or system involvement M32.8: other forms of systemic lupus erythematosus

### Screening, Informed Consent, Randomization, Data Collection, and Blinding

Study information, including recruitment materials and screening survey links, is available via the study website [24], where interested individuals can click on a screening survey link to complete initial screening and eligibility verification. Participants complete online eligibility screening and e-consent through the online study informatics platform, called CHOIR [16], with health care use derived from linked electronic health record and claims data where available.

Study data are collected and managed using the CHOIR platform, with secure, password-protected storage and built-in data quality checks (eg, range and completeness checks) to support data integrity. Participant-reported level of depression symptoms are continuously monitored through the platform, with predefined clinical thresholds triggering alerts to study staff and escalation procedures, including follow-up and referral to appropriate care as needed.

**Figure 1:**
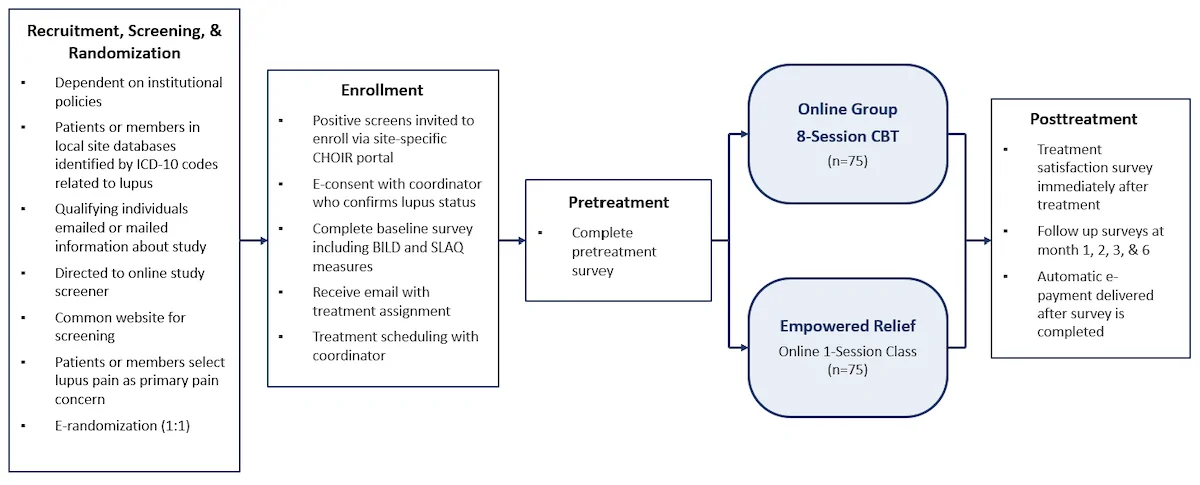
Recruitment methods and participant flow.

After completing the study screener to ensure initial eligibility, randomization occurs using CHOIR’s randomization functionality to ensure balanced 1:1 allocation (ie, 75 per arm). Informed consent takes place with a study coordinator via phone. However, group assignment (Empowered Relief or CBT) is only disclosed to participants after completion of the baseline survey. To maintain study integrity, treatment allocation remains concealed from investigators and biostatisticians until database closure after the 6-month follow-up, whereas site personnel and treatment providers necessarily remain unblinded for operational execution. Figure 1 details the study recruitment methods and participant flow.

The study is guided by a multilevel patient-centered engagement infrastructure, including a 76-member national patient advisory panel, a 17-member local patient advisory board, and a central 10-member patient engagement and diversity board with a patient representative with lupus, which collaboratively inform study design, recruitment strategies, patient-facing materials, and implementation processes to ensure accessibility and inclusivity [22]. These advisory boards prioritize diverse representation across demographic and lived experience domains and are actively engaged throughout the study life cycle [22]. Additionally, condition-specific advisory input contributes to tailoring study procedures and materials to enhance inclusivity for specific patient populations.

### Data Collection and Management

CHOIR [25] is used as the study informatics platform to confirm eligibility, obtain electronic consent, distribute automated emails, and collect self-report data. The study uses a hybrid workflow combining automated processes with strategic coordinator touchpoints developed in response to local regulatory requirements and informed by patient advisor feedback, emphasizing the value of coordinator interaction for participant engagement and retention.

### Study Treatments

Lupus PROGRESS compares 2 evidence-based online skill-based pain treatments that have common and distinct elements and differ in treatment duration and intensity.

#### CBT for Chronic Pain

The CBT protocol is Learning About My Pain (LAMP), a literacy-adapted, manualized curriculum developed by Thorn et al [26-29]. Doctoral-level pain psychologists trained on LAMP will deliver it over 8 weekly 2-hour sessions (16 total hours) via a videoconference platform and using an electronic slide deck. The eight session topics are (1) “Pain and Stress,” (2) “How the Brain Deals With Pain and How to Influence the Brain,” (3) “Getting Active and Other Useful Health Habits,” (4) “Pain and Emotions,” (5) “Pain and Communication,” (6) “Recognizing and Managing Thoughts That Work Against You,” (7) “Working with Deeper Beliefs,” and (8) “Reviewing Your Pain Coping Toolbox.” The structured curriculum emphasizes practical skill building within a collaborative group environment where 12 to 15 participants share experiences, receive peer and therapist validation, build supportive connections, and receive tools and directions for home-based practice. Participants who miss the initial session are reassigned to new cohorts; later absences do not trigger rescheduling. Camera use is encouraged but optional, with equipment provided as needed. LAMP fidelity rating forms are completed by site coordinators collaborating with therapists, with oversight by the CBT director [9,29].

#### Empowered Relief

Empowered Relief is a 1-session (2 hours) didactic and experiential class that is delivered by certified clinician instructors (trained through Stanford continuing medical education workshops). The intervention involves pain neuroscience education, content on the connection between stress and pain, self-regulation skills drawn from cognitive behavioral and mindfulness approaches [30], an experiential exercise, self-assessment, completion of a personalized plan for ongoing daily use of the skills, and a free binaural guided relaxation app (Calm Tool) that participants download onto their smartphones or personal devices for ongoing and unlimited use. The highly structured content is delivered via an electronic slide deck and instructor scripts. The didactic format requires no personal introductions or disclosure. Unlike the CBT group size requirement, Empowered Relief can accommodate any number of participants. Empowered Relief permits anonymous attendance with cameras disabled, and unlike with CBT, participants may invite a family member or other support people to attend the class with them. Materials are distributed in advance via email and mail. Study coordinators complete fidelity checklists during each class to ensure that the full treatment protocol is administered, and participants download the Calm Tool app and learn how to use it.

### Study Aims and Hypotheses

The study primary aims were selected based on patient preference surveys administered in the parent PROGRESS study.

Primary aim 1 is to compare treatment groups for multiple primary outcomes (pain intensity and pain interference) at 3 months after treatment. We hypothesize that Empowered Relief will be noninferior to 8-session CBT for reducing pain intensity or pain interference.

Primary aim 2 is to compare treatment groups for 4 priority secondary outcomes (sleep disturbance, pain catastrophizing, pain bothersomeness, and anxiety), treatment completion and adherence, and treatment burden. We hypothesize that Empowered Relief will be noninferior to 8-session CBT for improving priority secondary outcomes at 3 months after treatment and that Empowered Relief will have superior treatment completion and adherence (number and percentage of those attending Empowered Relief or at least 5 sessions of CBT) and lower burden ratings than 8-session CBT.

Primary aim 3 is to compare treatment groups for other secondary outcomes (satisfaction with social roles and responsibilities, patient global impression of change, anger, fatigue, and depression). We hypothesize that Empowered Relief will be noninferior to CBT for improving secondary outcomes at 3 months after treatment.

Primary aim 4 is to compare the durability of treatment effects at 6 months after treatment. We hypothesize that Empowered Relief will be noninferior to 8-session CBT for multiple primary outcomes and priority secondary outcomes at 6 months after treatment.

The exploratory aim is to compare health care use and opioid use through electronic health record and Centers for Medicare & Medicaid Services (CMS) data 3 months prior to enrollment to the last 3 study months. We will assess medication status (eg, stable dose, voluntary tapering, or involuntary tapering), as well as medication access (eg, no access problems or difficulty with medication access). We hypothesize that both groups will show reduced use during the final 3 study months.

We will also investigate the trajectory of both treatments individually (within-group treatment outcomes).

### Study Measures

Self-reported demographic data are collected at baseline and include age, gender identity, sex at birth, race and ethnicity, household composition, educational attainment, veteran status, disability status, employment status, annual household income, and pregnancy status. Additional baseline variables include pain history, prior behavioral pain treatment, current pain treatments, and barriers to pain care. Substance use is assessed using the National Institute on Drug Abuse 4-item quick screen, pain distribution is assessed using the Stanford CHOIR visual body map [31], treatment expectancy is assessed after treatment assignment is revealed to participant using the 6-item Stanford Expectations of Treatment Scale [32], and immediately after treatment, satisfaction and perceived utility are assessed using study-specific items [9].

Participants complete validated measures at baseline; immediately before treatment; and at 1, 2, 3, and 6 months after treatment, with Table 1 showing each outcome, the specific measure used, and the time points of administration. Additional posttreatment measures include treatment burden and satisfaction; engagement is assessed via session attendance. Health care use is evaluated using electronic medical record or claims data comparing the 3 months prior to enrollment with the final 3 months of follow-up.

**Table 1:** Primary, priority secondary, other secondary, and exploratory outcomes.

Outcome type	Name of outcome	Specific measure to be used	Time points
Multiple primary	Pain intensity	Numeric rating scale (0-10) [33-36]	Baseline; immediately before treatment^a^; and months 1, 2, 3, and 6 after treatment
Multiple primary	Pain interference	PROMIS^b^ 6-item Pain Interference scale [36-41]	Baseline; immediately before treatment; and months 1, 2, 3, and 6 after treatment
Priority secondary	Sleep disturbance	PROMIS 6-item Sleep Disturbance scale [42]	Baseline; immediately before treatment; and months 1, 2, 3, and 6 after treatment
Priority secondary	Pain bothersomeness	Numeric rating scale (0-10)	Baseline; immediately before treatment; and months 1, 2, 3, and 6 after treatment
Priority secondary	Pain catastrophizing	Pain Catastrophizing Scale (13 items) [43,44]	Baseline; immediately before treatment; and months 1, 2, 3, and 6 after treatment
Priority secondary	Anxiety	PROMIS 6-item Anxiety scale [45-47]	Baseline; immediately before treatment; and months 1, 2, 3, and 6 after treatment
Secondary	Satisfaction with social roles and responsibilities	PROMIS 6-item Satisfaction With Social Roles and Activities scale [47]	Baseline; immediately before treatment; and months 1, 2, 3, and 6 after treatment
Secondary	Patient global impression of change	PROMIS 1-item Global Impression of Change scale [48-50]	Months 1, 2, 3, and 6 after treatment
Secondary	Depression	PROMIS 6-item Depression scale [51,52]	Baseline; immediately before treatment; and months 1, 2, 3, and 6 after treatment
Secondary	Fatigue	PROMIS 6-item Fatigue scale [53-56]	Baseline; immediately before treatment; and months 1, 2, 3, and 6 after treatment
Secondary	Anger	PROMIS 5-item Anger scale [57]	Baseline; immediately before treatment; and months 1, 2, 3, and 6 after treatment
Exploratory	Health service use	Number of medical visits during the final 3 mo of the study vs 3 mo prior to enrollment	Chart review or CMS^c^ data (Humana)
Exploratory	Lupus disease burden	Brief Index of Lupus Damage (28 items); numeric score [58]	Baseline and months 3 and 6 after treatment
Exploratory	Systemic lupus activity	Systemic Lupus Activity Questionnaire (24 items); numeric score [59]	Baseline and months 3 and 6 after treatment

^a^Administered if more than 2 weeks elapsed since baseline.

^b^PROMIS: Patient-Reported Outcomes Measurement Information System.

^c^CMS: Centers for Medicare & Medicaid Services.

SLE-specific measures include the Brief Index of Lupus Damage (BILD) [58], a validated patient-reported and dichotomous (yes or no) measure of cumulative organ damage in SLE, and the Systemic Lupus Activity Questionnaire (SLAQ) [59], a patient-reported instrument that uses a mixed-response format to assess lupus disease activity and recent symptom burden. The BILD assesses cumulative organ damage across 12 organ systems, capturing damage attributable to SLE that has been present for at least 6 months since disease onset. Each item is recorded as present or absent, yielding total scores ranging from 0 to 30, with higher scores reflecting greater accumulated organ damage. The SLAQ comprises 24 items assessing symptoms and signs of disease activity (eg, fatigue, fever, cutaneous manifestations, and arthritis) over the preceding 3 months. Each item is rated on a 4-point scale (“no problem,” “mild,” “moderate,” or “severe”), corresponding to scores from 0 to 3. Total scores range from 0 to 44, with higher values indicating greater SLE-related disease activity. Both measures are administered at baseline and at 3 and 6 months after treatment.

Harms are defined as adverse events, including participant-reported distress or other negative experiences, and are assessed through both structured study assessments and spontaneous participant report, with ongoing monitoring by study staff. Adverse events will be summarized by comparator and included in the final trial report.

### Power and Analytic Plan

Table 2 describes the study power to assess noninferiority across multiple primary outcomes, priority secondary outcomes, and other secondary outcomes at 3 months after treatment.

**Table 2:** Noninferiority margin and power.

Outcome		Time point	NM^a^ (SD for 3-month change)	NM in terms of the baseline (SD of the NM)	Power (%)
**Multiple primary outcomes**					
	Pain intensity	Month 3	2.8 (7.7) [60]	0.21 (7.3)	69
	Pain interference^b^	Month 3	4.5 (7.9) [39]	0.47 (6.4)	96
**Priority secondary outcomes**					
	Sleep disturbance^b^	Month 3	4.0 (6.2) [61]	0.16 (9.3)	99
	Pain bothersomeness	Month 3	1.5 (2.2) [62]	0.68 (2.2)	>99
	Pain catastrophizing	Month 3	6.8 (9.8) [63]	0.35 (11.3)	>99
	Anxiety^b^	Month 3	3.0 (7.0) [64]	0.33 (9.1)	89
**Other secondary outcomes**					
	Satisfaction with roles and responsibilities^b^	Month 3	2.0 (8.0) [61]	0.25 (8.0)	58
	Patient global impression of change^b^	Month 3	0.5 (1.5) [65]	0.33 (1.5)	75
	Depression^b^	Month 3	3.0 (7.6) [64]	0.29 (10.4)	85
	Fatigue^b^	Month 3	4.0 (9.6) [52]	0.43 (9.3)	88
	Anger^b^	Month 3	5.0 (10.9) [39]	0.43 (11.6)	92
**Exploratory outcomes**					
	Brief Index of Lupus Damage	Month 3	—^c^	—	—
	Systemic Lupus Activity Questionnaire	Month 3	—	—	—

^a^NM: noninferiority margin.

^b^These are Patient-Reported Outcomes Measurement Information System (PROMIS) measures that use T-scores (indicated by b; values represent <0.5 SD for each PROMIS variable).

^c^Not applicable.

The sample size of 75 patients per group was selected to support noninferiority testing for 2 primary outcomes (pain intensity and pain interference) at a 1-sided significance level of 10%/2=5%, and all secondary outcomes (sleep disturbance, pain bothersomeness, pain catastrophizing, anxiety, satisfaction with social roles and responsibilities, patient global impression of change, anger, fatigue, and depression) at a 1-sided significance level of 10%. Different significance levels were selected to account for multiple testing and reflecting the priority of the outcomes. Table 2 shows the power for the primary, priority secondary, and other secondary outcomes, with sufficient power (>70%) achieved for most outcomes except pain intensity, and satisfaction with roles and responsibilities; the study is not powered to detect changes in SLE-related activity and disease burden outcomes.

Primary analyses will evaluate the noninferiority of Empowered Relief compared with 8-session CBT for pain intensity and pain interference at 3 months after treatment based on estimated treatment effects from a mixed model for repeated measures (MMRM) with an unstructured covariance matrix under an intention-to-treat framework. We will conduct 2 separate MMRMs analyses for pain intensity and pain interference; for addressing missing data, MMRM is considered superior over other options such as multiple imputation or least observation carried forward. Specifically, treatment effects at month 3 will be estimated through the treatment-by-time interaction at the 3-month time point. Secondary analysis will similarly evaluate the noninferiority of Empowered Relief compared with CBT for 4 priority secondary outcomes and 5 other secondary outcomes at the month 3 visit. We will also evaluate the noninferiority of all the aforementioned outcomes at the month 6 visits. Exploratory analyses will evaluate within-group change trajectories and compare treatment completion, patient burden, and exploratory health care use outcomes between the 2 groups. Exploratory analyses will examine changes in the BILD and SLAQ for both groups.

Power calculations for multiple primary end points, priority secondary measures, and additional outcomes are shown in Table 2; adequate statistical power was achieved for general pain outcomes, although lupus-specific measures are exploratory given sample constraints. Enrolling 150 participants provides sufficient power to assess noninferiority between treatments for pain intensity and interference (multiple primary outcomes) applying a 1-sided α of .05 and priority secondary end points applying a 1-sided α of .10.

The primary analytic strategy tests whether Empowered Relief demonstrates noninferiority to CBT on pain intensity and interference at the 3-month primary end point. We will use MMRMs with unstructured variance-covariance matrices, analyzing all participants as randomized (intention to treat). Between-group differences will be quantified via treatment-by-time interaction terms evaluated at 3 months after the intervention.

Additional analyses address several secondary objectives: persistence of treatment gains at the 6-month follow-up, longitudinal symptom trajectories within each intervention arm, rates of treatment engagement and participant-reported burden, and patterns of health care service use. SLE-specific end points, including BILD [58] and SLAQ [59], will also be examined to characterize how interventions perform within this disease population, although these analyses are exploratory.

Exploratory heterogeneous treatment effect subgroup analyses will assess whether treatment effects differ by pain type, number of pain conditions, race and ethnicity, socioeconomic status, disability status, and insurance type—factors associated with pain burden and treatment access. Analyses will use the Tukey method for multiple comparison adjustment.

For both groups, we will summarize data on treatment burden, satisfaction, and completion; online ease; and missing data. We will examine effects by site, therapist, and insurance type. We will also examine CBT attendance as a continuous variable and baseline predictors for attrition.

### Ethical Considerations

Lupus PROGRESS is conducted in compliance with all participating site institutional review boards, with the coordinating approval provided by the Stanford University School of Medicine Institutional Review Board (65439). The study is conducted in accordance with the Declaration of Helsinki and adheres to Patient-Centered Outcomes Research Institute regulatory standards [66]. The study is overseen by an independent data safety and monitoring board meeting twice annually and reported in accordance with SPIRIT (Standard Protocol Items: Recommendations for Interventional Trials) guidelines and sponsor regulatory standards. The study has been registered on ClinicalTrials.gov (NCT05612750).

Participant identification is protected with a unique study identification number. All data will be received electronically, immediately locked in the database, and stored with double password protection. The project manager and treatment instructors will be unblinded to individual group assignment; coinvestigators will be blinded until 6-month data are received. Statisticians will remain blinded to group assignment until the database is locked. Participant blinding is not possible given the obvious difference in treatment duration (1 vs 8 sessions), with treatment assignment revealed only after completion of the baseline survey. In advertisements and consent forms, both treatments are described as expert-led evidence-based group treatments for chronic pain that involve learning, experiences, pain relief skills, and creating a personalized plan for pain relief.

Participants may discontinue their participation in the study at any time in response to distress, adverse experiences, or personal preference, with study staff available to provide support as needed. For 8-session CBT, personal information may be shared among the group. At the start of each treatment cohort, the instructor explains group confidentiality, requests that members adhere to it, and also outlines the “honor system” limits of confidentiality. A similar, briefer explanation is provided at the outset of Empowered Relief given its brief time frame and limited opportunities to share personal information. Participants assigned to Empowered Relief may take part anonymously if they wish.

All study participants may receive up to US $195 for completing all study assessments.

## Results

As of May 2026, we have enrolled 130 participants. Funding was received in April 2023. Enrollment is anticipated to end in November 2026, with treatments completed by April 2027 and full analyses completed by February 2028. Results are expected to be published in fall 2029.

## Discussion

The Lupus PROGRESS study seeks to compare 2 scalable online chronic pain interventions in a lupus-specific national randomized comparative effectiveness trial. The study design and selected outcomes were deeply informed by patient stakeholders, ensuring that the future evidence generated will be meaningful to the population. As both study treatments are delivered online, we expect that results will provide clinical evidence to inform accessible, low-risk, and home-based pain care for patients who have limited options for pain relief. The pragmatic study design prioritizes real-world applicability: accepting self-reported lupus diagnoses when verification is unavailable, centralizing CBT classes with patients without SLE for operational efficiency, and incorporating patient advisor input throughout the study. The inclusion of Humana as a study site allows us to conduct national, targeted study advertising to patients who have an SLE diagnosis, thus facilitating a geographically diverse study sample. Findings are expected to inform pain treatment matching and shared decision-making for patients with SLE, clinicians, and family members. Owing to the nationwide and diverse enrollment, we expect to recruit a representative sample and generate evidence that is generalizable to patients with lupus broadly. One limitation is that the study is powered at less than 80% for 3 outcomes.

Dissemination plans include written plain-language results summaries posted on the Patient-Centered Outcomes Research Institute website (sponsor) and communicated directly to study participants and advisors. Results will be published open access in peer-reviewed scientific journals and distributed broadly among patient advocacy groups.

## Appendix

Multimedia Appendix 1 Peer-review report by the Patient-Centered Outcomes Research Institute (PCORI), through a PCORI Project Program Award to Stanford University.

## Abbreviations

BILD: Brief Index of Lupus Damage
CBT: cognitive behavioral therapy
CMS: Centers for Medicare & Medicaid Services
LAMP: Learning About My Pain
MMRM: mixed model for repeated measures
PROGRESS: Pain Relief With Online Groups That Empower Skill-Based Symptom Reduction
SLAQ: Systemic Lupus Activity Questionnaire
SLE: systemic lupus erythematosus
SPIRIT: Standard Protocol Items: Recommendations for Interventional Trials
