# Supplementary material for: Empowered Relief vs Cognitive Behavioral Therapy for Patients With Systemic Lupus Erythematosus and Chronic Pain: Study Protocol for a National, Randomized, Noninferiority Trial
Source: JMIR Res Protoc. 2026 Aug 25;15:e100104. doi: 10.2196/100104 (PMC13554953; doi:10.2196/100104)
Supplement: Supplementary file 1 [file resprot_v15i1e100104_app1.docx]

**Appendix. Summary of Changes Following Merit Review**

1.      We provided standardization of the CBT protocol (selecting Dr. Beverly Thorn’s LAMP low literacy 8-session CBT protocol for the study), to be used across the sites.

2.     We detailed how the diverse populations of this study would be recruited through a detailed advisor engagement plan, subsequently outlined in the following two publications:

Monson EA, Mardian A, Saxena K, Dorsonne B, Hailu AY, Heggan E, Riazi G, Slater K, Poupore-King H, Clifton J, Perez L, Tian L, Porter J, Denton S, Hanson ER, Miefert J, Thorn BE, Mackey SC, Matthias C, Darnall BD. Randomized Comparative Effectiveness Study of 1-Session vs. 8-Session Online Behavioral Treatment for Chronic Pain: Protocol for the national PROGRESS study. *Pain Med* 2026. Feb 19. pnag027, <https://doi.org/10.1093/pm/pnag027>

Clifton J, Adair E, Cheung M, Torres C, Andrews W, Dorsonne B, Hailu A, Heggan E, Miefert J, Riazi G, Dildine TC, Spears S, Greer-Smith R, Pin T, Williams N, Perez L, King HP, Ziadni MS, Mackey S, Darnall BD. PROGRESS: A Patient-centered Engagement Infrastructure and Multi-level Approach to Enrich Diversity, Equity, and Inclusion in a National Online Behavioral Pain Treatment Study. *J Pain* special issue; Oct 23, 2024: <https://www.jpain.org/article/S1526-5900(24)00694-1/fulltext>

3. We added gender as a stratification factor and this will be coded in CHOIR and monitored by the Informatics and Data Core.

4. We added to the EHR data extraction prescription medications and specifically opioids.

5. The study team will explore dissemination through AARP and many other national advocacy groups. We anticipate a major public dissemination effort.

6. We included detailed methods for suicidal ideation screening, monitoring and triaging.

7. We justified the sample size and non-inferiority margin.
